# Novel Insights into Adipogenesis from the Perspective of Transcriptional and RNA N6‐Methyladenosine‐Mediated Post‐Transcriptional Regulation

**DOI:** 10.1002/advs.202001563

**Published:** 2020-09-03

**Authors:** Tongxing Song, Yang Yang, Siwen Jiang, Jian Peng

**Affiliations:** ^1^ Department of Animal Nutrition and Feed Science College of Animal Science and Technology Huazhong Agricultural University Wuhan 430070 China; ^2^ The Cooperative Innovation Center for Sustainable Pig Production Wuhan 430070 China; ^3^ Key Laboratory of Animal Genetics Breeding and Reproduction Ministry of Education College of Animal Science and Technology Huazhong Agricultural University Wuhan 430070 China

**Keywords:** adipogenesis, obesity, RNA m^6^A modification, transcription factors

## Abstract

Obesity is a critical risk factor causing the development of metabolic diseases and cancers. Its increasing prevalence worldwide has aroused great concerns of the researchers on adipose development and metabolic function. During adipose expansion, adipogenesis is a way to store lipids as well as to avoid lipotoxicity in other tissues, and may be an approach to offset the negative metabolic effects of obesity. In this Review, the transcriptional regulation of adipogenesis is outlined to characterize numerous biological processes in research on the determination of adipocyte fate and regulation of adipogenic differentiation. Notably, one of the post‐transcriptional modifications of mRNA, namely, N^6^‐methyladenosine (m^6^A), has been recently found to play a role in adipogenesis. Here, the roles of m^6^A‐related enzymes and proteins in adipogenesis, with a particular focus on how these m^6^A‐related proteins function at different stages of adipogenesis, are mainly discussed. The Review also highlights the coordination role of the transcriptional and post‐transcriptional (RNA m^6^A methylation) regulation in adipogenesis and related biological processes. In this context, a better understanding of adipogenesis at both the transcriptional and post‐transcriptional levels may facilitate the development of novel strategies to improve metabolic health in obesity.

## Graphical abstract

This work provides an overview on the roles of m^6^A‐related enzymes and proteins in adipogenesis at different stages, and highlights the coordinating effect of transcriptional and post‐transcriptional (RNA m^6^A methylation) regulation on adipogenesis. A better understanding of adipogenesis at both the transcriptional and the post‐transcriptional levels may facilitate the development of novel strategies to fight against obesity.

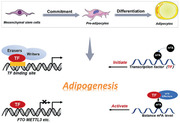

## 1 Introduction

The global epidemic of obesity has always been increasing for the past 30 years. As judged by the body mass index, nearly 40% of the adult population are overweight and about 13% are deemed as obese.^[^ ^1^ ^]^ It has been well established that obesity is correlated with a wide range of comorbidities such as insulin resistance and type 2 diabetes mellitus, nonalcoholic fatty liver, and dyslipidemia.^[^ ^2^ ^]^ Hence, the epidemic of obesity highlights the importance of elucidating the mechanism of adipose development, particularly adipogenesis.

Over the past three decades, the mechanisms of adipogenesis have been extensively studied, and adipogenesis has been found to be regulated by a complex transcriptional and epigenetic cascade.^[^ ^3^ ^]^ The “‘master regulator”’ of adipogenesis is peroxisome proliferator‐activated receptor (PPAR*γ*), which is both necessary and sufficient for fat cell formation.^[^ ^4^ ^]^ PPAR*γ* and CCAAT‐enhancer‐binding proteins (C/EBP*α*) work together with other transcription factors to activate the target genes required for terminal adipogenic differentiation. More and more key transcription factors have been identified to regulate adipogenesis both in vivo and in vitro.^[^ ^5^ ^]^ A series of excellent Reviews have provided details on the transcriptional regulation of adipogenesis.^[^ ^6^, ^7^, ^8^ ^]^ Notably, it has been shown that mRNA N^6^‐methyladenosine (m^6^A) modification functions as a novel and critical post‐transcriptional modulator of gene expression, which may be a new perspective to further and better understand adipogenesis.^[^ ^9^, ^10^, ^11^ ^]^

This Review aims to outline the mechanisms of adipogenesis under transcriptional and RNA N6‐methyladenosine‐mediated post‐transcriptional regulation. Special emphasis will be placed on the novel role of mRNA m^6^A modification in adipogenesis and important proteins related to post‐transcriptional regulation based on recent advances in the understanding of m^6^A. Finally, the Review will also discuss the potential of therapeutically targeting the activity of m^6^A‐related enzymes for the reduction of obesity.

## 2 Physiology of Adipose Tissues

Adipose tissues are crucial for systemic metabolic homeostasis. Originated from different subcompartments of the mesoderm, adipose tissues have various distributions mainly in subcutaneous and visceral depots in the body,^[^ ^12^, ^13^ ^]^ and exert distinct functions due to different compositions of fat cell types, namely, white, brown, and beige adipocytes. White adipocytes, which make up the bulk of white adipose tissue (WAT), are capable of storing chemical energy formatted as unilocular lipid droplets.^[^ ^13^ ^]^ By contrast, brown and beige adipocytes are characterized by the capability of dissipating energy to generate heat.^[^ ^14^, ^15^ ^]^ Characteristically, these adipocytes have abundant mitochondria, small lipid droplets and high uncoupling protein 1expression in human and mouse, while they can also be discriminated with high confidence by specific lineage as well as different depots. The detailed information about brown and beige adipogenesis has been well summarized in other excellent Reviews.^[^ ^14^, ^15^, ^16^, ^17^ ^]^ In this Review, we will focus on the adipogenesis of white adipocytes.

## 3 Molecular Mechanisms of Adipogenesis

### 3.1 Adipogenesis

**Figure 1:**
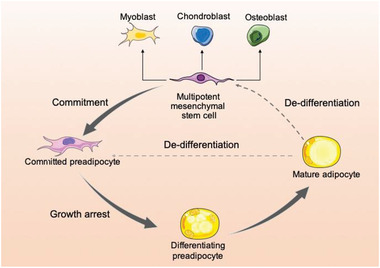
Overview of adipogenesis. Mesenchymal stem cells are also capable of forming adipocytes, myoblasts, osteoblasts, and chondroblasts. Adipogenesis is the process by which fibroblast‐like progenitor cells restrict their fate to adipogenic lineage (preadipocytes, also called adipogenic progenitor cells or adipogenic stem cells), finally differentiating to form adipocytes. There is now evidence showing that adipocytes can dedifferentiate back to fibroblast‐like progenitor cells in response to metabolic environments. The dedifferentiated cells can proliferate and redifferentiate to become adipocytes.

Studies of WAT development have been mostly focused on the cellular mechanism of adipocyte differentiation, which is termed as adipogenesis and refers to the process that fibroblast‐like progenitor cells (mesenchymal stem cells) differentiate into new adipocytes (**Figure** **1**).^[^ ^3^, ^6^, ^8^ ^]^ Adipogenesis is required by normal adipose tissues for mechanical protection and thermal insulation, and is characterized as a way to support WAT to store excess calories and adapt to increased energy expenditure. Hence, the newly formed fat cells are used to effectively sequester lipids to prevent lipotoxicity in other nonadipose tissues such as liver, muscle, heart, and placenta.^[^ ^8^ ^]^ For energy balance, WAT undergoes hypertrophy (size) and hyperplasia (number) of mature adipocytes in long‐term energy storage.^[^ ^13^ ^]^ From the point of view of tissue and cell biology, WAT is a complex organ composed by heterogeneous cells, primarily adipocytes, preadipocytes, vascular endothelial cells, and immune cells. Through a process called adipogenesis, adipocytes are derived from mesenchymal stem cells (MSCs) that have the capacity to develop into several cell types (adipocytes, osteocytes, chondrocytes, myocytes).^[^ ^3^ ^]^ At cellular level, with a process of commitment, the precursor cells become adipocyte lineages, which are called preadipocytes. Next, preadipocytes undergo several rounds of mitotic clonal expansion (MCE) and finally differentiate into adipocytes. Thus, the adipogenesis process comprises two main phases: commitment from MSCs to preadipocytes and terminal differentiation into mature adipocytes^[^ ^6^ ^]^ (Figure 1).

**Figure 2:**
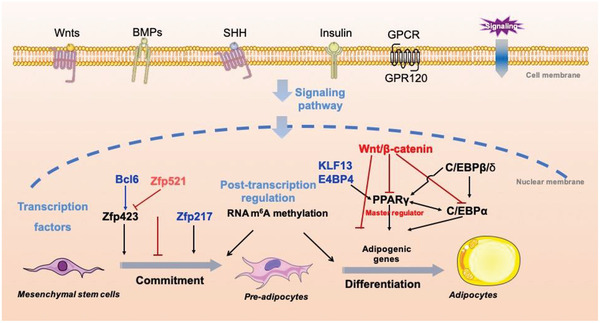
Regulation of adipogenesis. Adipogenesis is orchestrated by a complex of regulation cascades. When receiving a number of extracellular signals such as Wnts and BMPs, transcription factors, epigenetic and post‐transcriptional regulators are activated, promoting or inhibiting adipogenesis.

Clarification of the detailed mechanism of adipogenesis is still given top priority in current studies of adipose development and related metabolic diseases. A large number of transcription factors, coactivators/corepressors, epigenomic factors, and signaling pathways are involved in adipogenesis. The transcriptional cascade that promotes adipogenesis has also been studied in depth, and again, the most detailed information is focused on the transcription factors and signaling pathways that promote or repress adipogenesis (**Figure** **2**).^[^ ^13^, ^18^ ^]^ The specific roles of epigenetic factors implicated in histone modification and chromatin remodeling in adipogenesis have also been well documented by some excellent Reviews.^[^ ^7^, ^19^, ^20^, ^21^ ^]^ Here, we will focus on the main transcription factors and signaling pathways involved in the two phases of adipogenesis.

### 3.2 Roles of Transcription Factors in Adipogenesis

Numerous studies based on the prevalent cell model in vitro C3H10T1/2 and 3T3‐L1 have facilitated the faithful mimicking of the functional characteristics of adipogenesis. C3H10T1/2 line, which was established in 1973, has been well identified as a pluripotent line for studying the first phase of adipogenesis (commitment),^[^ ^22^ ^]^ while another cell line, 3T3‐L1 preadipocyte cell line, has become the “gold standard” for investigating the second phase of adipogenesis (terminal differentiation).^[^ ^6^, ^23^ ^]^ Likewise, the primary stromal vascular fractions (SVFs) isolated from human and other animal fat tissues have become an ex vivo way to mimic adipose development.^[^ ^24^ ^]^ Meanwhile, in vivo models involving mice with knockout or conditional knockout of certain genes are employed to overcome the drawbacks of in vitro studies and define the phenotypes observed from cell lines. In our group, by using piglets as a model, SVFs isolated from porcine adipose tissue and muscle treated with an adipogenic cocktail medium for adipogenesis were used to screen differential expression genes.^[^ ^25^ ^]^ Furthermore, some important transcription factors have been well characterized. Currently, some Reviews have summarized the progress in the research on adipose tissues and lipid metabolism,^[^ ^8^, ^13^ ^]^ and clarified the transcriptional cascade of adipocyte differentiation.^[^ ^7^, ^26^ ^]^ Here, we will highlight the main regulators during commitment and terminal differentiation that are known to date.^[^ ^27^, ^28^ ^]^

#### 3.2.1 Transcription Factors Regulating Commitment

To dissect the mechanism of adipogenic commitment, in vivo and in vitro models have been employed for adipogenic research. However, due to the complexity of the regulation cascade in commitment, some original and pendent questions remain to be answered, such as how MSCs are restricted into preadipocytes.^[^ ^29^ ^]^ Gupta et al. (2010) established adipogenic and nonadipogenic Swiss 3T3 fibroblast cell lines for screening vital factors of adipogenic commitment. It was observed that zinc finger protein 423 (*Zfp423*) was preferentially enriched in adipogenic fibroblasts (preadipocytes) in comparison with in nonadipogenic cell lines,^[^ ^30^, ^31^ ^]^ indicating that *Zfp423* plays an important role in the commitment stage of adipogenesis. Consistent with the in vitro studies of function loss and gain, whole body knockout of *Zfp423* resulted in smaller adipose tissues at embryo day 18.5.^[^ ^30^ ^]^ These findings may help to mark *Zfp423* as a critical transcription factor for commitment.^[^ ^31^ ^]^ Notably, to find out the upstream regulators of *Zfp423*, a novel transcription factor, B cell lymphoma 6 (*BCL6*), has been reported and characterized by our group.^[^ ^32^ ^]^ Knockdown of *BCL6* was shown to reduce the expression of *Zfp423*. By RNA sequencing and promoter analysis, *BCL6* was found to enhance the promoter activity of signal transducer and activator of transcription 1, and was identified as an upregulator of *Zfp423*, early B cell factor 1 (*Ebf1*), and PPAR*γ*.^[^ ^32^ ^]^ However, in vivo model of conditional knockout of *BCL6* in adipose tissues showed that *BCL6* deletion would lead to WAT expansion,^[^ ^33^ ^]^ implying that *BCL6* possibly plays different roles in regulating early adipogenesis and late lipogenesis. From some RNA‐seq data used to identify *BCL6*, we have also identified a novel transcription factor *Zfp217* that regulates early adipogenesis of C3H10T1/2 cell line^[^ ^25^, ^34^ ^]^ (Figure 2), implying that the transcriptional atlas of commitment can be further improved. In addition, another group identified a repressor of adipogenesis from zinc finger protein family, namely, *Zfp521*,^[^ ^35^ ^]^ which acts as a paralog of *Zfp423* and inhibits commitment by binding to *Ebf1* to block the expression of *Zfp423* (Figure 2). Some other groups have also identified some new negative regulators of commitment, such as transcription factor 7‐like 1.^[^ ^36^ ^]^

Although a number of transcription factors have been documented, determination of how progenitor cells acquire a preadipocyte commitment remains quite challenging.^[^ ^37^ ^]^ Much of our knowledge regarding the molecular mechanisms of adipogenic commitment is based on cell culture models involving fibroblast‐like cells (both primary cells and cell lines) that differentiate to form adipocytes in vitro. However, there is no single consensus marker or well defined markers of preadipocytes in vivo, reflecting the high heterogeneity of adipose progenitor cells.^[^ ^8^, ^38^, ^39^ ^]^ Mice lineage‐tracking system and single‐cell sequencing have revealed heterogeneity among preadipocytes, providing novel insights into the mechanism of adipogenesis^[^ ^40^, ^41^, ^42^ ^]^ (reviewed extensively elsewhere^[^ ^38^, ^39^ ^]^).

#### 3.2.2 Transcription Factors Regulating Terminal Differentiation

By taking advantages of cell lines and traditional adipogenic cocktail medium, terminal differentiation has been better clarified than commitment. As mentioned above, PPAR*γ* is characterized as the master regulator of adipogenesis,^[^ ^4^, ^43^ ^]^ because of its indispensable role in adipocyte differentiation in vitro^[^ ^4^ ^]^ and in vivo.^[^ ^44^ ^]^ Even though a large number of regulators have been identified during commitment and terminal differentiation, they are finally dependent on the expression and/or activity of PPAR*γ* during adipogenesis (Figure 2). It has also been found that mouse PPAR*γ* has two truncated isoforms. Notably, PPAR*γ*1 is widely expressed in several tissues and cell types, including adipose tissue, skeletal muscle, liver, and bone, whereas PPAR*γ*2 expression is limited almost exclusively to adipocytes.^[^ ^45^ ^]^ In fact, PPAR*γ*2 has more adipogenic potential than PPAR*γ*1, and is essential for effective adipogenesis in vitro.^[^ ^4^, ^43^ ^]^ Expression of mouse PPAR*γ*2 is induced during the differentiation of cultured adipocyte cell lines and is strikingly adipose‐specific in vivo, which was identified as the first adipocyte‐specific transcription factor in mouse.^[^ ^43^ ^]^ We have previously reported that eicosapentaenoic acid upregulates the expression of PPAR*γ*1 by activating RXRa, and then PPAR*γ*1 binds to the functional peroxisome proliferator responsive element in the promoter of adipocyte‐specific PPAR*γ*2 to continuously activate the expression of PPAR*γ*2 throughout the transdifferentiation process.^[^ ^46^ ^]^ PPAR*γ*1 enhances the expression of PPAR*γ*2 during transdifferentiation of mouse myoblast cell line C2C12 into adiponectin‐expressed cells, namely, adipocytes.^[^ ^46^ ^]^ To fully activate the adipogenic differentiation, PPAR*γ* and its most important downstream effector C/EBP*α* functionally synergize^[^ ^47^ ^]^ and bind to more than 90% of the same DNA binding sites to activate the transcription of genes expressed in mature adipocytes, such as fatty acid binding protein 4 (FABP4, also called aP2) and adiponectin.^[^ ^48^ ^]^

However, the expression levels of C/EBP*α* and PPAR*γ* are low in the early process of adipogenesis, indicating that there are earlier modulators which regulate these transcription factors. C/EBP*β* is another member of C/EBP family acting as an activator of PPAR*γ* and C/EBP*α* transcription, which has transient high expression during the early stage of adipogenesis. It is evident that the regulatory factors targeting C/EBP*β* are essential for the development of adipose tissues, and play a pivotal role in multiple rounds of MCE (Figure 2).^[^ ^49^ ^]^ Another class of factors, namely, the kruppel‐like transcription factor (Klf) family, were shown to have high expression in early adipogenesis. The members including Klf4, Klf5, and Klf15 promote adipocyte differentiation, which is on the contrary inhibited by Klf2, Klf3, and Klf7.^[^ ^50^ ^]^ Klf4 can bind to the promoter of C/EBP*β* to induce the expression of PPAR*γ* and Klf15. Likewise, another member of KLF family, Klf13, directly binds to the promoter of PPAR*γ* to activate its expression.^[^ ^51^ ^]^ In addition, Klf5 is regulated by the glucocorticoid receptor (GR).^[^ ^52^ ^]^ Our group has identified E4 promoter‐binding protein 4 (E4BP4, also called NFIL3), which was significantly induced by glucocorticoid (Dexamethasone) via GR,^[^ ^53^ ^]^ indicating that E4BP4 and Klf15 might be the targets of the cocktail medium to promote adipogenesis.^[^ ^54^ ^]^ These results indicate that some hormone‐related signals such as E4BP4‐GR axis might help to regulate PPAR*γ* from another perspective. Targeting these key transcription factors may facilitate a better understanding of the regulation of adipogenesis by transcriptional cascades (Figure 2).

### 3.3 Main Pathways and Receptors in Adipogenesis

In addition to the key regulatory factors, several signaling hormones and ligands have also been shown to be involved in adipogenesis (Figure 2).

#### 3.3.1 Wingless and INT‐1 (Wnt) Signaling

Wnt signaling, for example, is perhaps the first characterized suppressor of adipogenesis.^[^ ^55^, ^56^ ^]^ During the commitment of MSCs, it was observed that Wnt signaling plays a role in commitment from C3H10T1/2 to A33 preadipocytes. R‐spondins‐2 and ‐3, which are activators of Wnt signaling, showed higher expression in committed A33 preadipocyte cell line. These findings indicate that Wnt signaling functions in the early commitment of adipogenesis.^[^ ^6^ ^]^ Canonical Wnt signaling results in the stabilization of *β*‐catenin. In preadipocytes, this stabilization leads to a failure of PPAR*γ* and C/EBP*α* expression and, in some cases, a shift toward an osteoblastic or other cell phenotypes.^[^ ^55^, ^57^, ^58^, ^59^ ^]^ In late adipogenic program, ablation of Wnt signaling promotes the expression of PPAR*γ*, causing transdifferentiation of myoblasts into adipocytes in vitro.^[^ ^55^ ^]^ Wnt may also redirect adipocyte dedifferentiation, implying that it is an intrinsic suppressor for both the induction and maintenance of adipose phenotype.^[^ ^60^ ^]^

#### 3.3.2 Transforming Growth Factor‐*β* (TGF‐*β*)/Bone Morphogenetic Protein (BMP) Signaling

TGF‐*β* signaling also acts as a potent inhibitor of adipogenesis.^[^ ^61^ ^]^ TGF‐*β*1 is a secreted protein that binds to TGF receptors, leading to the activation of SMAD proteins that induce the expression of extracellular matrix related genes such as collagen proteins to restrict adipogenesis by inhibiting PPAR*γ*.^[^ ^61^, ^62^ ^]^ However, other molecules in the TGF‐*β* super family called BMPs, which were originally defined for their functions in cartilage and bone formation, have recently been shown to play different roles from TGF‐*β* in adipogenesis.^[^ ^63^, ^64^, ^65^ ^]^ Interestingly, members of BMPs have distinct functions to promote adipogenesis of white and brown adipocytes.^[^ ^63^, ^64^, ^65^, ^66^ ^]^ BMP2 and BMP4, for example, have been identified as potent proadipogenic factors that drive adipogenesis at the early stage in vitro, whereas BMP7 plays a crucial role in promoting the adipogenesis and thermogenesis of brown adipocytes.^[^ ^63^, ^65^, ^66^ ^]^ Notably, both Wnt and BMP signaling are involved in maintaining the development of various organs, implying that their functions at different stages may be spatiotemporally regulated.

#### 3.3.3 Insulin Signaling

As a pancreatic peptide hormone, insulin plays a predominant physiological role in glucose metabolism, and functions to bind the insulin receptor or the related insulin‐like growth factor 1 (IGF1) receptor. The insulin signaling cascade activates insulin receptor substrates and then PI3K/AKT kinases, leading to the activation of downstream CREB, mTOR, and the family of forkhead proteins (FOXOs).^[^ ^67^, ^68^ ^]^ It is widely acknowledged that these intracellular effectors in insulin signaling are inevitably associated with nutritional physiology and metabolism, and elimination of them blocks adipogenesis.^[^ ^69^, ^70^, ^71^, ^72^, ^73^, ^74^, ^75^, ^76^, ^77^ ^]^ Thus, insulin is an essential component of adipogenic differentiation medium in vitro and promotes adipogenesis in highly adipogenic progenitor cells by FACS.^[^ ^40^ ^]^ Even though glucocorticoid signaling has been reported to sensitize preadipocytes to insulin signaling and consequently enhance adipogenic action of the insulin pathway,^[^ ^78^ ^]^ it still needs to be clarified how other signals facilitate or suppress insulin signaling.^[^ ^8^ ^]^

#### 3.3.4 G‐Protein‐Coupled Receptors (GPCRs)

Many eicosanoids signal via cell surface GPCRs to regulate adipogenesis, which has also been well clarified. Long chain fatty acids (LCFAs) function as the ligands of PPAR*γ*, which is famous for its role as a nuclear receptor, as well as the ligands of specific GPCRs.^[^ ^27^, ^79^, ^80^ ^]^ Treatment with LCFAs may not only activate PPAR*γ*, but also activate the membrane fatty acid receptor GPR120 and subsequently enhance the expression of PPAR*γ* by the ERK1/2‐Ca^2+^ pathway.^[^ ^27^, ^81^, ^82^ ^]^

Some other pathways such as Notch^[^ ^83^, ^84^, ^85^, ^86^ ^]^ and hedgehog^[^ ^87^, ^88^ ^]^ signaling may also play critical roles in adipogenesis, which have not been well characterized.^[^ ^60^ ^]^ These pathways may help to expand our sights into the combating against obesity and related metabolic diseases. In short, the regulatory mechanism of adipogenesis by transcriptional cascades has been well manifested as illustrated in Figure 2.

## 4 Post‐Transcriptional Regulation: RNA m^6^A Modification in Adipogenesis

Although DNA sequence conveys the majority of heritable information to subsequent generations, accumulating evidence demonstrates that epigenetics plays an important role in regulating cell‐type‐specific gene expression during cell differentiation.^[^ ^89^, ^90^ ^]^ Epigenetics includes several different mechanisms, such as DNA methylation, histone modification, and chromatin remodeling.^[^ ^19^, ^91^, ^92^ ^]^ However, RNA can be also modified, which can be a key biomarker for several biological events.^[^ ^11^ ^]^ For instance, N^6^‐methyladenosine (m^6^A), a common modification in mRNA, has been detected in 1970s and its roles in regulating gene expression are being uncovered.^[^ ^11^ ^]^

### 4.1 Overview of RNA m^6^A Modification

More than 150 distinct chemical modifications of RNAs have been found to date, and m^6^A has been identified as the most abundant modification on eukaryotic messenger RNA (mRNA).^[^ ^93^ ^]^ m^6^A modification is widely distributed in viruses, bacteria, yeast, plants, and vertebrates.^[^ ^94^, ^95^, ^96^, ^97^ ^]^ In 2011, Jia et al. discovered the first demethylase, the fat mass, and obesity‐associated protein (FTO), confirming that m^6^A modification is dynamic and reversible.^[^ ^98^ ^]^ Subsequently, m^6^A RNA IP sequence (MeRIP‐seq) was established based on specific m^6^A antibody immunoprecipitation,^[^ ^99^, ^100^ ^]^ which leads to numerous studies of RNA epigenomics and makes it one of the research hotspots. Further work showed that m^6^A modification is highly conserved in a sequence context as RRm^6^ACH (R represents G and A; H represents A, C, and U),^[^ ^101^ ^]^ and is preferentially distributed around stop codons and enriched at 3 ‘UTR, 5‘UTR, and the inner exons.

**Figure 3:**
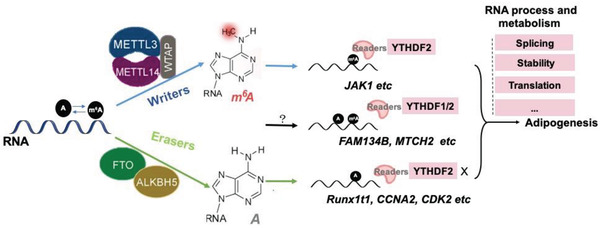
Impact of m^6^A on mRNA abundance in adipogenesis. The “writer”‐methyltransferase complexes METTL3, METTL14, and WTAP; “eraser”‐demethylase FTO and ALKBH5; “reader”‐m^6^A binding proteins YTH family and IGF2BPs affect m^6^A methylation and RNA metabolism of key genes (such as *Runx1t1*) to regulate adipogenesis.

Among the effectors of m^6^A, three types of key proteins are known to be involved in balancing m^6^A modification: “writer”‐methyltransferases, “eraser”‐demethylases, and “reader”‐m^6^A binding proteins. m^6^A modification is deposited to RNAs by a core methyltransferase complex composed of METTL3 and METTL14 heterodimer, in which METTL3 acts as the catalytic subunit and METTL14 facilitates RNA binding. Another key protein WTAP, which binds to METTL3/14, is required for optimal substrate recruitment and METTL3/14 localization.^[^ ^93^ ^]^ The “eraser”‐demethylases comprise demethylases FTO and alkB homolog 5 (ALKBH5) to remove the methylation. The “reader”‐m^6^A binding proteins include YTHs, HNRNPs, and IGF2BPs, which recognize m^6^A and help to define biological events by post‐transcriptional regulation of mRNA^[^ ^11^, ^102^ ^]^ (**Figure** **3**).

m^6^A modification has been shown to regulate nearly every stage of mRNA processing, including mRNA processing and metabolism (export, splicing, decay, translation)^[^ ^103^ ^]^ (Figure 3). Based on the understanding of its dynamic reversibility and widespread roles in mRNA metabolism, m^6^A modification was reported to play important roles in numerous physiological processes, such as animal development, gametogenesis, T cell homeostasis, stress responses, circadian rhythm, cell differentiation, and reprogramming, and is regarded as a post‐transcriptional regulatory mechanism.^[^ ^10^, ^103^, ^104^, ^105^ ^]^

Researchers have also focused on the function of m^6^A modification in adipogenesis^[^ ^9^, ^28^, ^106^ ^]^ and obesity‐related events. m^6^A was shown to be responsive to high‐fat treatment in mice.^[^ ^107^ ^]^ Another group set up an obese porcine model (Landrace pigs as the lean‐type breed and Jinhua pigs as the obese‐type breed) to identify the unique peaks of m^6^A in mRNA using m^6^A sequencing.^[^ ^108^ ^]^ These discrepant m^6^A distributions in vivo revealed that the variation of m^6^A modification may be related to adipose function and adipogenesis, and m^6^A‐related proteins play a specific and important role in modulating adipogenesis. Hence, we summarize recent studies to present the new molecular regulatory mechanisms of m^6^A on adipogenesis.

### 4.2 m^6^A “eraser” FTO Promotes Adipogenesis

A genome‐wide association analysis has identified the roles of FTO in obesity and related diseases.^[^ ^109^, ^110^ ^]^ A number of studies have reported the positive function of FTO in fat mass, body weight, and adipogenesis.^[^ ^111^, ^112^ ^]^ The m^6^A‐dependent role of FTO was first reported by Zhao et al. (2014), as the global m^6^A level was increased during adipogenesis of mouse 3T3‐L1 cells. Knockdown of FTO impaired adipogenesis, and overexpression of catalytically inactive FTO did not restore adipogenic differentiation.^[^ ^106^ ^]^

#### 4.2.1 FTO Functions as a Cell Fate Regulator for Bone Marrow Stromal Cells (BMSCs) to Promote Adipogenic Commitment

FTO participates in GDF11‐FTO‐PPAR*γ* axis to control the shift of osteoporotic BMSCs fate to the formation of more adipocytes instead of osteoblasts.^[^ ^113^ ^]^ These results suggest that demethylase FTO and FTO‐mediated low m^6^A modification act as a switch to transform osteogenesis to adipogenesis.

#### 4.2.2 FTO Enhances Adipogenesis by MCE Progress

It has also been shown that FTO regulates m^6^A modification in the mRNA of *cyclin A2* (*CCNA2*) and *cyclin‐dependent kinase 2* (*CDK2*), which are crucial cell cycle regulators, leading to delayed entry of MDI‐induced 3T3L1 cells into G2 phase.^[^ ^114^ ^]^ It was found that FTO negatively regulates m^6^A levels and positively regulates adipogenesis in porcine preadipocytes.^[^ ^115^ ^]^ Furthermore, it has been found that FTO deficiency suppresses *Janus kinase 2* (*JAK2*) expression in an m^6^A‐dependent manner of porcine and mouse preadipocytes, leading to increases in the phosphorylation of *STAT3* and attenuating the transcription of C/EBP*β*, which inhibits adipocyte differentiation at the early MCE stage.^[^ ^116^ ^]^ Furthermore, it was obeserved that FTO functions as a regulator of alternative splicing regulatory protein Srsf2, and the identified runt‐related transcription factor 1 (*Runx1t1*) is the core target gene of FTO.^[^ ^106^ ^]^ Subsequent research revealed that FTO might exert its promoting effects on adipogenesis through enhancing the expression of the short isoform of RUNX1T1 at the early MCE stage.^[^ ^112^ ^]^

#### 4.2.3 Recently, FTO Was Found to Regulate Autophagy during Adipogenesis

m^6^A modification also plays a critical role in regulating autophagy and adipogenesis of mouse 3T3‐L1 and porcine primary preadipocytes through targeting Atg5 and Atg7. Knockdown of FTO decreased the expression of ATG5 and ATG7, leading to attenuation of autophagosome formation, thereby inhibiting autophagy and adipogenesis.^[^ ^117^ ^]^ These results provide a novel insight that m^6^A methylation regulates adipogenesis by targeting autophagy.

Collectively, increasing evidence supports that FTO‐dependent m^6^A demethylation plays a pivotal role in adipogenesis. FTO promotes adipogenesis partly by influencing MCE stage and autophagy, while other mechanisms for its adipogenesis‐promoting effect still need further elucidation. Even though a lot of studies have shown the role of demethylase FTO in adipogenesis, it still remains confusing how FTO protein itself is regulated. In the research on the upstream mechanism of FTO, our group has identified zinc finger protein (*Zfp217*) as a transcriptional regulator of FTO to enhance adipogenesis in an m^6^A‐dependent way, which provides a new perspective to understand the modulation of m^6^A modification during adipogenesis.^[^ ^28^ ^]^

### 4.3 m^6^A “Writer” METTL3 Represses Adipogenesis

Compared with FTO, METTL3 has an opposite function in adipogenesis. A recent study has reported that METTL3 deficiency appears to promote adipogenic differentiation in mouse 3T3L1 cells.^[^ ^106^ ^]^

METTL3 suppresses the commitment and MCE of adipogenesis in vitro. Interestingly, it was reported that the deletion of METTL3 causes inhibited bone formation and marrow fat accumulation. The loss of METTL3 compromised osteogenic potential and increased adipogenic differentiation through m^6^A RNA methylation of the critical regulator of lineage allocation in MSCs and osteoblast precursors.^[^ ^118^, ^119^ ^]^ Mechanically, knockdown of METTL3 decreased the m^6^A level of *JAK1* and increased its expression in porcine BMSCs, resulting in upregulated *STAT5* phosphorylation to bind to the promoter of C/EBP*β*.^[^ ^120^ ^]^ These findings uncover consistent molecular mechanisms of METTL3‐dependent m^6^A modification in JAK‐STAT‐C/EBP*β* pathway, and provide novel insights into the regulation of the early MCE stage of adipogenesis by m^6^A‐related proteins.

However, other research groups have identified that METTL3, METTL14, and WTAP positively control adipogenesis by promoting cell cycle transition in MCE of 3T3‐L1 cells.^[^ ^121^ ^]^ Consistent with the in vitro results, WTAP^+/−^ mice did not show diet‐induced obesity with smaller size and number of adipocytes, resulting in increased energy expenditure, attenuated hepatic steatosis, and macrophage infiltration and better insulin sensitivity.^[^ ^121^ ^]^ The observed complex and obscure regulatory effects of m^6^A modification on adipogenesis may be due to the efficiency of gene interference and complexity of in vivo study. Another view of point is that all these homozygous gene knockout mice are embryonic lethal, indicating the critical role of m^6^A writer in animal development.^[^ ^121^ ^]^ Thus, further research is needed to reveal the role of methyltransferase complex in adipogenesis.

To sum up, these results comprehensively reveal that demethylase FTO and FTO‐mediated low m^6^A modification act as a switch, whereas methyltransferase METTL3 has the opposite effect in transforming osteogenesis to adipogenesis. After methylation or demethylation, the alteration of m^6^A methylation by FTO or METTL3 is ultimately performed by recruiting specific “reader” proteins to m^6^A sites, which impact the fate of target mRNAs by influencing RNA metabolism.^[^ ^122^ ^]^

### 4.4 m^6^A “Readers” in Adipogenesis

#### 4.4.1 YTHs Mediate RNA Metabolism of Key Genes to Inhibit Adipogenesis

YT521‐B homology (YTH) domain family including YTHDF1–3 and YTHDC1–2 has been well identified as m^6^A readers. YTHDF1/3 were reported to promote the translation of target transcripts and YTHDF2 promotes the degradation of mRNA.^[^ ^123^ ^]^ Previous studies have revealed that the degradation of key mRNA mediated by YTHDF2 may be the most significant impact of m^6^A methylation on adipogenesis. After m^6^A modification is erased or deposited by FTO or METTL3, YTHDF2 increases or decreases the recognition and degradation of methylated mRNAs of *CCNA2, CDK2*, *JAK1/2*, *Atg5* or *Atg7*, which consequently alters the expression of target proteins and adipogenic differentiation.^[^ ^114^, ^116^, ^117^, ^120^ ^]^ Moreover, mRNA modification on single genes was also explored. Loss of m^6^A in the mRNA of *FAM134B* was found to facilitate adipogenesis, and YTHDF2 may recognize and bind to its m^6^A sites, resulting in reduced mRNA stability and protein expression.^[^ ^124^ ^]^ It is worth noting that YTHDF2 maintains m^6^A methylation by limiting the activity of demethylase FTO under heat shock stress,^[^ ^125^ ^]^ providing more potential ways for the “reader” proteins to participate in adipogenesis. Consistently, we also found that *Zfp217* interacts with YTHDF2 to maintain the m^6^A‐demethylation activity of FTO to promote adipogenesis.^[^ ^28^ ^]^

In addition to the degradation of mRNA by YTHDF2, YTHDF1 also regulates adipogenesis through increasing protein expression. It was found that m^6^A positively mediates *patatin‐like phospholipase domain containing 2* expression and functions by promoting translation most likely through YTHDF1.^[^ ^108^ ^]^ Meanwhile, the research group has also revealed the role of m^6^A methylation of key genes in adipogenesis of intramuscular preadipocytes. They identified a unique methylated gene in Jinhua pigs, *mitochondrial carrier 2*, which promotes intramuscular adipogenesis in an m^6^A‐YTHDF1‐dependent manner.^[^ ^126^ ^]^

Besides YTHDF1/2, other YTH domain family proteins may also have functions in adipogenesis. Nuclear m^6^A reader YTHDC1 functions as a recruiter of mRNA splicing factors, and ZNF638 pre‐mRNA is a key target.^[^ ^127^ ^]^ It is known that ZNF638 physically interacts and transcriptionally cooperates with C/EBP*β*/*δ* and splicing regulators to promote adipogenesis.^[^ ^128^, ^129^ ^]^ These findings imply that YTHDC1 may affect adipogenesis partly through ZNF638, which is worthy of further study.

Overall, YTH family functions as a downstream regulator of mRNA methylation and metabolism to mediate adipogenesis.

#### 4.4.2 Other m^6^A “Readers” in Adipogenesis

In addition to YTH family, some RNA binding proteins may also be m^6^A “readers” in adipogenesis. IGF2BPs, a distinct family of m^6^A readers, could maintain the stability and storage of mRNAs depending on m^6^A modification.^[^ ^130^ ^]^ It was shown that overexpression of IGF_2_BP1 promoted adipocyte differentiation and lipid droplet accumulation of chicken preadipocytes, whereas inhibition of IGF2BP1 showed the opposite effect.^[^ ^131^ ^]^ These results suggest that IGF_2_BPs promote adipogenesis in an m^6^A‐dependent manner and the target mRNAs of IGF2BPs deserve further research.

Human antigen R (HuR, also called ELAVL1), a well‐established RNA‐binding protein, binds to the U‐rich regions at the 3′‐UTR of thousands of transcripts to increase the mRNA stability and translation efficiency of the target genes.^[^ ^54^ ^]^ HuR also serves as a potential m^6^A reader as identified by an RNA affinity chromatography approach.^[^ ^99^ ^]^ Knockdown of METTL3 or METTL14 enhanced the binding of HuR to demethylated mRNA, which subsequently increased RNA stability in embryonic stem cells.^[^ ^132^ ^]^ It has been reported that HuR binds to the mRNA of C/EBP*β*, which is required for the onset of adipogenesis,^[^ ^133^, ^134^ ^]^ suggesting that it is a new way for HuR to act as an m^6^A “reader” to regulate adipogenesis.

Therefore, the functional outcome of m^6^A modification in regulating adipogenesis depends on the reading of specific m^6^A sites of transcripts by m^6^A “readers” to influence their RNA metabolism. These studies highlight the potential regulatory mechanisms by which m^6^A “readers” regulate adipogenesis in an m^6^A‐dependent manner.

## 5 Orchestration of Transcriptional and Post‐Transcriptional Regulation of Adipogenesis

In central dogma of molecular biology, mRNA plays a key role of connecting link between the preceding and the following. DNA is transcribed into mRNA, which is then translated into protein. Around mRNA, transcription factors recognize specific DNA sequences as “master regulators,” performing the first step of decoding genetic information.^[^ ^135^ ^]^ A series of transcription factors along with DNA epigenetic regulators constitute a complex and orchestrated transcriptional cascade to regulate gene expression. In addition, post‐transcriptional regulators, especially m^6^A modification, affect almost every stage of mRNA processing to alter gene expression at the post‐transcriptional level, which controls adipogenesis and many other processes.^[^ ^136^ ^]^

To regulate gene expression, both transcrptional and post‐transcrptional regulation show spatiotemporal continuity. 1) The occurrence of post‐transcriptional modification depends on the initiation of transcription. 2) Then, the post‐transcriptional modification influences the translation of transcripts through participating in RNA processing and metabolism. 3) Finally, post‐transcriptional regulation could facilitate rapid remodeling of the transcriptome relative to transcriptional regulation.^[^ ^137^ ^]^

Thus, to clarify the coordination of transcriptional and post‐transcriptional regulation may be a critical and profound topic in current studies of adipogenesis.

### 5.1 m^6^A‐Related Proteins Modulate Adipogenesis through Transcriptional Regulation

#### 5.1.1 The Transcription of Key Regulators Is Regulated by m^6^A Modification to Mediate Adipogenesis

**Figure 4:**
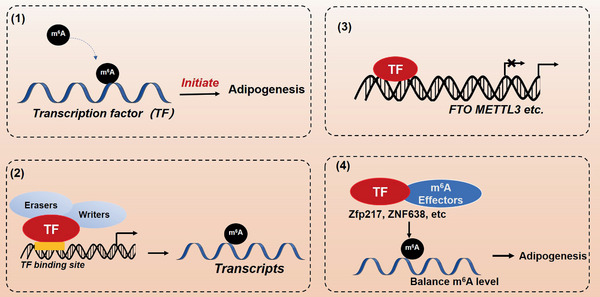
Complex regulation at transcriptional and post‐transcriptional levels by m^6^A modification in adipogenesis. (1) m^6^A‐related proteins regulate the transcription of key regulators to mediate adipogenesis in an m^6^A modification‐dependent manner. (2) m^6^A‐related proteins play a direct role in transcriptional regulation. (3) m^6^A‐related proteins may be transcriptionally regulated. (4) Besides transcriptional regulation, transcription factors interact with m^6^A‐related proteins to regulate m^6^A methylation.

In many pivotal biological events, m^6^A modification occurs in the mRNAs of some key transcription factors. The dynamic changes of methylated or demethylated mRNAs may alter the cellular levels of transcripts and proteins to affect cell fate^[^ ^137^ ^]^ (**Figure** **4**). In adipose tissues, researchers have revealed a potential relationship between transcriptional regulation and post‐transcriptional modification using MeRIP‐Seq.^[^ ^138^ ^]^ These results reveal the potential mechanism by which m^6^A‐related proteins regulate the mRNA methylation of vital transcription factors.

During adipogenesis, the methylation of the master regulator, PPAR*γ*, has been reported recently.^[^ ^113^ ^]^ It was observed that FTO facilitates the differentiation of BMSCs into adipocytes rather than osteoblasts.^[^ ^113^ ^]^ Furthermore, FTO binds to and demethylates the mRNA of PPAR*γ*, and consequently enhances its expression. In FTO‐knockdown BMSCs, high m^6^A modification in the mRNA of PPAR*γ* inhibited the expression of PPAR*γ*, suggesting a negative role of m^6^A in adipogenesis.^[^ ^113^ ^]^ These results suggest that the regulation of PPAR*γ* by m^6^A modification may be one way besides transcription and activation of PPAR*γ* to regulate adipogenesis.

Another key regulator in adipogenesis, C/EBP*β*, has also been found to be regulated by m^6^A modification.^[^ ^116^ ^]^ FTO deficiency inhibited the expression of *JAK2* and phosphorylation of *STAT3*, leading to decreased expression of C/EBP*β*,^[^ ^116^ ^]^ while knockdown of METTL3 decreased the mRNA m^6^A level of *JAK1*, resulting in enhanced mRNA stability of *JAK1*, and activated phosphorylation of *STAT5* to bind to the promoter of C/EBP*β* in the adipogenic differentiation of pig BMSCs.^[^ ^120^ ^]^ Thus, C/EBP*β* may not be the direct target of RNA m^6^A modification in BMSCs, which has also been demonstrated in 3T3‐L1 cell line by some groups.^[^ ^139^ ^]^ In our previous study, RNA m^6^A modification was also not detected in the mRNA of C/EBP*β* in 3T3‐L1 cell line with or without adipogenic induction.^[^ ^28^ ^]^ Thus, m^6^A might modulate the upstream regulators of key transcription factors to inhibit adipogenesis.

C/EBP*α* has been identified to be regulated by FTO in other biological events but not in adipogenesis, implying that C/EBP*α* might also be a target of m^6^A modification in adipogenesis.^[^ ^140^ ^]^ Thus, it may be speculated that m^6^A modification in adipogenesis has multiple functions to directly modify some key transcription factors or modulate their upstream key regulators. Although some potential mechanisms have been identified to be related to m^6^A modification, further studies are needed to more comprehensively reveal the functions of RNA m^6^A methylation in the regulation of adipogenesis.

#### 5.1.2 m^6^A‐Related Proteins Bind the DNA Sequence of Target Genes to Regulate Their Transcription

The nuclear localization of m^6^A effectors indicates that they may play different roles in different cell types and/or in response to different stimuli and stresses. Demethylase FTO has been well known for its role in obesity and related diseases and can act as a transcription cofactor. FTO is abundant in both the nucleus and cytoplasm of certain cell lines, but mostly in nucleus.^[^ ^141^ ^]^ Interestingly, FTO is recruited to both unmethylated and methylated promoters and enhances the transactivation potential of the C/EBPs to modulate the transcriptional regulation of adipogenesis.^[^ ^142^ ^]^ Furthermore, FTO was also reported to demethylate DNA N6‐methyldeoxyadenosine (6mA), and affect the transcriptional activation of C/EBP*δ* by demethylating 6mA at the promoter of C/EBP*δ* in adipogenesis.^[^ ^139^ ^]^

For methyltransferases, METTL3/14‐WTAP is distributed in nucleus to bind the DNA sequence of target genes to regulate their transcription. It was observed that METTL3 and/or METTL14 are located in the relevant chromatin sites, which co‐occurs with accumulated m^6^A methylation.^[^ ^143^, ^144^ ^]^ METTL3/14‐WTAP is recruited at desired chromatin loci, and cotranscriptionally regulates m^6^A installation most likely with transcription factors and histone marks.^[^ ^93^ ^]^ Besides METTL3/14‐WTAP, PCIF1, a cap‐specific m^6^A methyltransferase, interacts with the C‐terminal domain of RNA polymerase II, suggesting that PCIF1 may perform its function in a cotranscriptional manner.^[^ ^145^ ^]^ Likewise, it was found that transcription factors SMAD2 and SMAD3 recruit the methyltransferase complex (METTL3/14‐WTAP) and promote its binding to a subset of transcripts (i.e., NANOG) involved in early cell fate, which reveals the connection between transcriptional and post‐transcriptional regulation.^[^ ^146^ ^]^ These findings suggest that methyltransferases can also function as transcriptional regulators during biological events. It would be interesting to further illustrate the function of methyltransferases in transcriptional regulation of adipogenesis.

Collectively, these findings unravel that FTO and methyltransferase complex may be implicated not only in m^6^A modification, but also in transcriptional regulation directly (Figure 4).

### 5.2 m^6^A‐Related Proteins May Be Transcriptionally Regulated

Although numerous studies have reported that m^6^A‐related proteins such as METTL3, FTO, and YTHDF2 play important roles in regulating gene expression and adipogenesis, little is known about the upstream mechanism by which these m^6^A‐related proteins themselves are regulated (Figure 4). We previously reported an interesting mode of transcription and post‐transcription of gene expression in adipogenesis.^[^ ^28^ ^]^ It was found that deficiency of transcription factor *Zfp217* inhibited adipogenesis in 3T3L1 cells and led to a global increase in m^6^A mRNA methylation. However, *Zfp217* is neither a methyltransferase nor a demethylase. It remains unclear how *Zfp217* inhibits the mRNA methylation of m^6^A. We subsequently demonstrated that *Zfp217* acts as a transcription factor and binds to the promoter of FTO gene to regulate its expression and promte adipogenesis.^[^ ^28^ ^]^ Thus, these results reveal a pivotal mechanism to combine gene transcription with m^6^A mRNA modification, which may be a novel way to modulate m^6^A modification during adipogenesis and other vital events.

Notably, it was demonstrated that there is a feedback regulatory mechanism between transcriptional and post‐transcriptional level in R‐2‐hydroxyglutarate (R‐2HG) mediated anti‐leukemic activity.^[^ ^140^ ^]^ R‐2HG exhibited a repressing effect on FTO demethylation activity, thereby increasing m^6^A methylation of the mRNA of key transcription factor C/EBP*α*, which would inhibit the expression of C/EBP*α* and leukemia cell proliferation/viability. Interestingly, low protein levels of C/EBP*α* in turn decreased mRNA level of FTO due to transcriptional regulation to enhance m^6^A modification. The feedback regulation between transcriptional and post‐transcriptional level provides some implications for dissecting the molecular mechanism of adipogenesis, and ultimately combating obesity and its associated risks of metabolic syndromes.

In addition, it was reported that METTL3 may be regulated by post‐transcriptional and post‐translational regulations. It has been shown that methyltransferase METTL3 could be influenced by miRNAs and SUMOylation,^[^ ^147^, ^148^ ^]^ and METTL3/14 complex is phosphorylated at several sites which might be functionally important.^[^ ^149^ ^]^

### 5.3 Besides Transcriptional Regulation, Transcription Factors Interact with m^6^A‐Related Proteins to Regulate m^6^A Methylation

Once a protein is identified as a transcription factor, the DNA binding domain and transcriptional activation domain will be well characterized. However, other key protein domains might be often ignored. It is recognized that transcription factors exert their functions through interacting with other proteins, suggesting their multifunctional roles in biological events (Figure 4). Notably, *Zfp217* is known as a core component of protein complexes with histone deacetylase (HDAC), CoREST, and CtBPs,^[^ ^150^, ^151^, ^152^ ^]^ and interacts with m^6^A methyltransferase METTL3 in embryonic stem cells (ESCs).^[^ ^105^ ^]^ *Zfp217* suppresses METTL3 activity, preventing the deposition of m^6^A on core transcripts to regulate pluripotency and reprogramming of ESCs. In regulation of adipogenesis, it was also validated that there is an actual interaction between *Zfp217* and m^6^A “reader” protein YTHDF2. *Zfp217* could act as a regulator of YTHDF2‐FTO competition, which sequesters YTHDF2 from limiting the accessibility of FTO to specific m^6^A sites.^[^ ^28^ ^]^

Interestingly, it was found that transcriptional coregulator ZNF638 interacts with another m^6^A “reader” HNRNPA2B1 in adipogenesis,^[^ ^129^ ^]^ suggesting that ZNF638 may function in an m^6^A‐dependent manner. Therefore, these studies shed light on the novel role of transcription factors to orchestrate gene transcription and m^6^A RNA modification, which offers a new approach for dissecting the molecular mechanism of adipogenesis.

## 6 Therapeutic Implications for Obesity‐Related Metabolic Disorders

Given that m^6^A‐related proteins play critical roles in adipogenesis, they may be potential therapeutic targets of adipogenesis and obesity‐related metabolic disorders. For example, the classical methylation inhibitor, i.e., cycloleucine, can significantly decrease the mRNA m^6^A level in a dose‐dependent manner in porcine adipocytes and increase intracellular triglyceride, whereas the methyl donor betaine shows the opposite effect.^[^ ^115^ ^]^ Epigallocatechin gallate (EGCG), a component in green tea, can effectively prevent obesity and the prevalence of type 2 diabetes mellitus.^[^ ^153^ ^]^ Wu et al. found that EGCG reduces the expression of FTO, leading to an overall increase in levels of RNA m^6^A methylation and ultimately inhibiting adipogenesis by blocking the MCE at the early stage.^[^ ^154^ ^]^ Although the above substances have been found to affect adipogenesis through RNA m^6^A methylation, their functions and effects remain to be validated in vivo.

**Figure 5:**
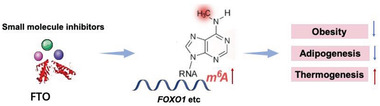
Potential inhibitors of FTO to regulate obesity‐related homeostasis.

Importantly, small‐molecule inhibitors have been developed to inhibit the catalytic activity of m^6^A effectors to combat obesity and cancer. Recently, virtual screening approach was employed to find FTO inhibitors from FDA‐approved drugs; as a result, entacapone was identified as a chemical inhibitor of FTO.^[^ ^155^ ^]^ Entacapone inhibits FTO demethylation activity, which thereby increases the energy expenditure and improves glucose tolerance of mice. FTO deficiency regulates FOXO1 expression by demethylating m^6^A sites, and entacapone was confirmed to regulate gluconeogenesis and thermogenesis through FTO–FOXO1 axis in vivo (**Figure** **5**). FB23 and FB23‐2 were reported to directly bind to FTO and selectively inhibit the m^6^A demethylase activity of FTO. FB23‐2 significantly affects the proliferation, differentiation, and apoptosis of human acute myeloid leukemia cells in vitro and in vivo.^[^ ^156^ ^]^ These results suggest that small‐molecule inhibitors specifically targeting FTO could be developed for fighting against obesity‐related metabolic disorders and other events (Figure 5). The findings broaden our horizon that numerous regulatory molecules affecting m^6^A modification can serve as effective treatment strategies of obesity‐related metabolic disorders (summarized in **Table** **1**).^[^ ^157^, ^158^, ^159^, ^160^, ^161^, ^162^, ^163^, ^164^ ^]^

**Table 1:** Functions of compounds targeting m^6^A effectors in homeostasis

Regulator	Target protein	Effect	Primary functions	Refs.
Rhein et al.	FTO	Inhibit	Reversibly bind FTO enzyme and competitively prevent the recognition of m^6^A substrates	[158]
MO‐I‐500	FTO	Inhibit	Inhibit survival and/or colony formation of SUM149‐MA cells	[158, 160]
Meclofenamic acid (MA) and MA2, the ethyl ester form of MA	FTO	Inhibit	Inhibit glioblastoma stem cells tumorigenesis	[161, 162]
R‐2‐hydroxyglutarate (R‐2HG)	FTO	Inhibit	Antileukemic activity	[140]
Epigallocatechin gallate	FTO	Inhibit	Reduce the expression of FTO and adipogenesis	[154]
IOX3	ALKBH5	Inhibit	Inhibitor of ALKHB5	[163]
Citrate	ALKBH5	Inhibit	Modest inhibitor of ALKHB5	[164]

With regard to methyltransferase, there have been few reports about targeting METTL3 to regulate adipogenesis and combat obesity. It is known that METTL3 may also have other functions besides its catalytic activity in cancers, and cytoplasmic‐localized METTL3 was proposed as a potential m^6^A reader.^[^ ^93^, ^165^ ^]^ Thus, development of inhibitors to target special RNA m^6^A methyltransferase has good application prospects in future studies.

Overall, the relationship between transcriptional and post‐transcriptional regulation by m^6^A‐related proteins may provide some novel therapeutic targets for combating obesity.

## 7 Conclusion and Perspective

In summary, from the above Review, the transcriptional mechanisms of adipogenesis have been well studied in the past three decades, while there have been relatively fewer studies of the post‐transcription of adipocyte differentiation, particularly RNA modifications. In this context, we summarized the progress in the studies of m^6^A modification in adipogenesis and identified *Zfp217*, a pivotal modulator of transcriptional and post‐transcriptional regulation in adipogenesis, which may help to better explore the orchestrated regulation at different levels in adipocyte differentiation. Besides, the regulation model for gene expression that coordinates RNA m^6^A methylation and gene transcription may provide a new approach to better understand other biological events.

Despite of the rapid progress in the studies of transcriptional and post‐transcriptional regulation of adipogenesis, many questions remain to be answered.

First, although both in vitro cell lines and in vivo models have provided reliable methods to manifest the key regulators in adipogenesis, the detailed mechanism for commitment phase is still unclear. Recently, a group led by Patrick Seale applied scRNA‐seq and FACS to identify new surface markers for progenitor cells and committed preadipocytes in WAT, defining a mesenchymal cell hierarchy involved in adipocyte formation.^[^ ^8^, ^38^, ^40^ ^]^

Second, the role of m^6^A in adipogenesis needs to be better characterized. The roles of RNA processing and metabolism modulated by m^6^A need to be further demonstrated in adipogenesis, and more importantly in lipid metabolism, which has not been reported yet. To find the “classical” drugs from FDA system for targeting novel m^6^A‐related enzymes such as FTO^[^ ^10^, ^155^ ^]^ may facilitate a better application of these potential drugs to treat obesity. Answering these questions may promote our understanding of m^6^A‐dependent adipogenesis, provide new insights into adipogenesis, and develop new approaches to treat obesity, cancer, and other metabolic diseases.

## Conflict of Interest

The authors declare no conflict of interest.
